# Multiple species of wild tree peonies gave rise to the ‘king of flowers’, *Paeonia suffruticosa* Andrews

**DOI:** 10.1098/rspb.2014.1687

**Published:** 2014-12-22

**Authors:** Shi-Liang Zhou, Xin-Hui Zou, Zhi-Qin Zhou, Jing Liu, Chao Xu, Jing Yu, Qiang Wang, Da-Ming Zhang, Xiao-Quan Wang, Song Ge, Tao Sang, Kai-Yu Pan, De-Yuan Hong

**Affiliations:** 1 State Key Laboratory of Systematic and Evolutionary Botany, Institute of Botany, The Chinese Academy of Sciences, Beijing 100093, People's Republic of China; 2 School of Horticulture, Southwest University, Chongqing 400716, People's Republic of China

**Keywords:** conservation, hybridization, *Paeonia*, RNA-seq, species tree

## Abstract

The origin of cultivated tree peonies, known as the ‘king of flowers' in China for more than 1000 years, has attracted considerable interest, but remained unsolved. Here, we conducted phylogenetic analyses of explicitly sampled traditional cultivars of tree peonies and all wild species from the shrubby section *Moutan* of the genus *Paeonia* based on sequences of 14 fast-evolved chloroplast regions and 25 presumably single-copy nuclear markers identified from RNA-seq data. The phylogeny of the wild species inferred from the nuclear markers was fully resolved and largely congruent with morphology and classification. The incongruence between the nuclear and chloroplast trees suggested that there had been gene flow between the wild species. The comparison of nuclear and chloroplast phylogenies including cultivars showed that the cultivated tree peonies originated from homoploid hybridization among five wild species. Since the origin, thousands of cultivated varieties have spread worldwide, whereas four parental species are currently endangered or on the verge of extinction. The documentation of extensive homoploid hybridization involved in tree peony domestication provides new insights into the mechanisms underlying the origins of garden ornamentals and the way of preserving natural genetic resources through domestication.

## 1. Introduction

Agricultural and industrial developments have considerably reduced biodiversity, which in turn has threatened our sustainability [1,2]. The domestication of food crops that began approximately 10 000 years ago initiated agriculture and laid the foundation of the civilization [3], which also turned approximately one-tenth of the terrestrial ecosystem into cropland [4]. This has contributed at least partly to the reduction of biodiversity, including that of the wild progenitors of crops when the crops took over the habitats of their wild relatives [5,6]. Despite this well-documented impact, we know little about the consequences of ornamental plant cultivation. While humans exploit plant diversity for their own survival and better living, the impact is mutually important and the complexity of the interactions requires in-depth studies [2]. Understanding the origin of cultivated ornamentals and the impact of cultivation on plant diversity, especially their wild relatives, should add a much-needed component to our knowledge about human and biodiversity interactions.

Cultivated tree peonies, collectively named *Paeonia suffruticosa* Andrews, were recorded in cultivation in China 1400 years ago [7]. Because of their beauty and fragrance, cultivated tree peonies were crowned the ‘king of flowers' in the Chinese Tang Dynasty, symbolizing happiness, richness and prosperity. In the Song Dynasty, nearly 203 peony cultivars were described [8]. They are now popular in temperate gardens, and China alone has more than 1000 modern cultivars.

*Paeonia suffruticosa*, together with nine wild shrubby species, constitute section *Moutan* DC of the genus *Paeonia* L. in the family Paeoniaceae [9,10]. In contrast to the success of cultivars, the majority of the wild species have become rare or endangered. The wild populations of *P. cathayana* and *P. ostii* have been completely eradicated, and *P. qiui* is critically endangered. A few populations of *P. decomposita*, *P. jishanensis*, *P. ludlowii*, *P. rockii* and *P. rotundiloba* exist. Only *P. delavayi* is still relatively common.

The origin of cultivated tree peonies has attracted much attention, but remains unclear. Although interspecific hybridization was speculated to be a common mechanism giving rise to the cultivars [9,11–16], no convincing evidence is available to substantiate the origin of the cultivars, especially with regard to the number of wild species involved in hybridization. This study represents a comprehensive effort to investigate the origin of cultivated tree peonies with the complete sampling of all wild species and extensive sampling of traditional cultivars, together with multiple phylogenetic markers from both nuclear and chloroplast genomes. In addition to the goal of clarifying the origin of cultivars, we intended to understand the consequences of the cultivation, especially those related to diversity and conservation of the wild gene pool of section *Moutan*.

## 2. Material and methods

### (a) Material sampling

The total of 441 accessions from all 37 known populations of the nine wild tree peony species were previously collected in Anhui, Gansu, Henan, Hubei, Shaanxi, Shanxi, Sichuan, Xizang and Yunnan Provinces of China, and the genetic variations within and among these populations were evaluated [17,18]. On basis of this survey, 26 accessions from 24 populations of the wild tree peony species were sampled in this study (electronic supplementary material, table S1). Four accessions from *P. brownii* and *P. califonica* were used as outgroups. Although there are more than 1000 tree peony cultivars at the present time, the vast majority of them were bred since the 1950s, primarily by crossing traditional cultivars. Given our goal of understanding the origin of cultivated tree peonies, we included only traditional cultivars in this study. There are about 100 traditional cultivars currently preserved in the two major historical and modern centres of tree peony cultivation, Luoyang of Henan Province and Heze of Shandong Province of China. In this study, 47 accessions were sampled to represent the diversity of the traditional cultivars (electronic supplementary material, table S1).

### (b) Chloroplast gene data collection

Fast-evolving regions of the chloroplast genome were screened following Dong *et al.* [19] according to the nucleotide diversity per site (*π*), calculated using DnaSP v. 5 [20]. The survey identified 14 chloroplast regions, including the recommended plant DNA barcodes of *matK*, *rbcL* and *trnH-psbA*. Primers and PCR conditions followed Dong *et al.* [19] and Yu *et al.* [21]. The sequences of the amplified regions were determined using the Big-Dye Terminator method and an ABI 3730xl DNA Analyzer (Applied Biosystems, Foster City, CA, USA). The Chloroplast DNA sequences obtained from this study have been deposited in the GenBank database under accession numbers KJ945637–KJ946200.

### (c) Nuclear gene data collection

Winter buds were collected from an individual of *P. lactiflora* Pall, grown in the Beijing Botanical Garden for RNA isolation. RNA-seq was conducted on the Illumina/Solexa Genome Analyzer at Beijing Genome Institute in Shenzhen. BLASTN was used to identify scaffolds and contigs for potential genes. The sequences of potential genes were run on BLAST again to determine whether there were any introns in the potential genes. Primers were designed to amplify the introns of *P. ostii* with the lengths of 500–2500 bp, which were suitable for conventional PCR and cloning. When necessary, PCR fragments were cloned into plasmids and eight colonies were sequenced using the Big-Dye Terminator method. The isolates of a single type are considered from a single locus and the locus was further tested for its resolution. Eight samples were used to test the resolutions of the sequences and only the loci resolved on the maximum-parsimony (MP) trees were considered in the second round of screening using *P. ludlowii*. The procedures and the criteria for the second round of screening were similar to the first round. The differences were that 16–24 colonies were sequenced and 71 samples were used to test the resolutions of the sequences, and only the loci resolved on the MP trees were selected for use.

Primers specific to the selected loci were designed and used to amplify the wild and cultivated peony accessions. The amplified PCR products were sequenced directly for wild accessions because no sequence polymorphism was found. For cultivars, direct sequencing identified highly polymorphic sequences. Thus, the PCR products of the cultivated accessions were cloned into pGM T-easy vectors (TianGen Biotech, Beijing). For each accession of a cultivar, eight to 16 clones were selected for sequencing. The nuclear DNA sequences generated for this study have been deposited in the GenBank database under accession numbers KM092534–KM093728.

### (d) Data analysis

Both the chloroplast and nuclear sequences were aligned using ClustalX [22] and manually adjusted. Potential compositional bias from the heterogeneity of nucleotide compositions among lineages was tested using the *χ*^2^-test. In order to investigate potential intragenic recombination in our data, we used the RDP program [23] to examine the alignments. Six recombination detection methods (RDP, GENECONV, Chimaera, MaxChi, BootScan and SiScan) were implemented and the default settings were used.

Fourteen chloroplast regions were concatenated for the phylogenetic analysis of the wild species. For the study of the origins of the cultivars, the four chloroplast regions that were most variable among the wild species were sequenced and used as phylogenetic markers for analysing cultivars and wild species. For the nuclear sequences, the 25 loci were concatenated for phylogenetic analyses of the wild species. For analyses involving the cultivars, seven loci were analysed separately. The best-fit model of DNA evolution was determined by the Akaike information criterion in MrModeltest v. 2.3 [24].

The phylogenetic analyses were performed using the MP and maximum-likelihood (ML) methods with PAUP* v. 4.0b10 [25] and PhyML v. 3.0 [26], and Bayesian inference (BI) using MrBayes v. 3.1.2 [27]. The topological robustness was assessed by bootstrap values in the MP and ML analyses and by posterior probability in the BI analysis. In the BI analyses, two independent Markov chain Monte Carlo (MCMC) runs were initiated from random starting trees with four simultaneous chains, one cold and three incrementally heated. At least 10 000 000 generations were run until stationarity in Markov chains was achieved, and every 1000 generations were sampled with the first 20% samples discarded as burn-in. The convergence of the MCMC algorithm was examined through convergence diagnostic PSRF and Tracer v. 1.4 [28].

In addition to the approach of concatenating sequences from multiple genes, the species trees were inferred from *BEAST based on multispecies coalescent models [29]. This model emphasized incomplete lineage sorting as the main source of gene tree discordance, and the uncertainty in gene trees and other model parameters was accommodated in a Bayesian phylogenetic framework. In this study, *BEAST algorithm implemented in software package BEAST v. 1.7.0 was attempted for species tree estimation [28]. We used the Yule process as species tree prior and the Piecewise constant and linear model for population size modelling. Convergence of MCMC chain was explored by running at least two independent analyses and checked by Tracer v. 1.4.

## 3. Results

*De novo* assembly of the RNA-seq data of *P. lactiflora* resulted in 20 697 scaffolds ranging from 476 to 4891 bp, and 14 722 contigs ranging from 100 to 1558 bp. BLASTN identified 4910 candidate genes (electronic supplementary material, dataset S1), of which 566 genes contained introns of presumably suitable sizes for conventional PCR. After amplifying and sequencing 503 of these genes, 58 genes were found to be potentially single-copy. Introns of these genes that were larger than 200 bp were sequenced and examined for the phylogenetic information among the sampled wild species. Based on the results, variable intron markers from 25 genes that could be easily sequenced with the least sequence polymorphism within an individual were identified as nuclear markers. These markers ranged from 322 to 3656 bp in length, 3.6–18.8% in variable sites and 1.7–8.6% in phylogenetically informative sites among the sampled tree peony species (table 1). The results of the *χ*^2^-test showed that there was no significant heterogeneity of base composition (*p* = 0.777–1.000). The potential intragenic recombinants of nuclear sequences were excluded using the RDP program.

**Table 1.** The detailed information of the 29 fragments of the 25 nuclear genes and 14 fragments of chloroplast genome sampled in this study.

fragment no.	genome	transcriptone fragment	aligned length	variable sites (%)	parsimony informative sites (%)
1	nuclear	C406508	699	8.0	7.2
2	nuclear	C437844	905	5.1	4.1
3	nuclear	scaffold02625	455	5.7	5.1
4	nuclear	scaffold02983	1208	5.5	3.3
5	nuclear	scaffold03365	2326	5.0	4.4
6	nuclear	scaffold03436	838	8.2	5.4
7	nuclear	scaffold03856	860	10.2	8.3
8	nuclear	scaffold04312	497	4.6	4.0
9	nuclear	scaffold04453	711	8.4	7.7
10	nuclear	scaffold04832	376	5.9	4.5
11	nuclear	scaffold10501	1463	7.9	6.6
12	nuclear	scaffold12044	3656	9.2	7.0
13	nuclear	scaffold12186	1026	3.6	1.7
14	nuclear	scaffold14018	1672	6.4	3.2
15	nuclear	scaffold16442	571	11.7	8.6
16	nuclear	scaffold16545	776	9.3	7.7
17	nuclear	scaffold17482	496	18.8	6.7
18	nuclear	scaffold17974	761	10.9	4.1
19	nuclear	scaffold19740	322	5.0	2.8
20	nuclear	scaffold20144	779	7.6	5.3
21	nuclear	scaffold20297	1408	8.6	6.9
22	nuclear	scaffold20535	683	4.2	2.6
23	nuclear	scaffold20599	516	7.4	6.8
24	nuclear	scaffold20612	457	4.4	3.9
25	nuclear	scaffold20685	1060	11.4	8.0
	nuclear total		24 521	7.7	5.5
1	chloroplast	*accD-ycf4*	854	4.0	3.4
2	chloroplast	*atpH-atpI*	956	4.3	3.5
3	chloroplast	*matK*	585	3.2	1.2
4	chloroplast	*ndhC-trnV*	968	5.1	3.2
5	chloroplast	*ndhH-ycf1*	1806	3.9	3.0
6	chloroplast	*petD-rpoA*	930	4.1	2.5
7	chloroplast	*psbE-petL*	1782	2.5	1.6
8	chloroplast	*psbM-trnD&trnY-trnE*	938	4.8	3.9
9	chloroplast	*rbcL*	641	0.6	0.3
10	chloroplast	*ropC1*	947	2.5	1.6
11	chloroplast	*rpc16-rps3*	713	4.2	3.6
12	chloroplast	*trnH-psbA*	413	4.6	3.6
13	chloroplast	*trnK-rps16*	947	5.0	3.5
14	chloroplast	*ycf1-a*	935	6.6	5.2
	chloroplast total		13 415	3.9	2.8

**Figure 1.**
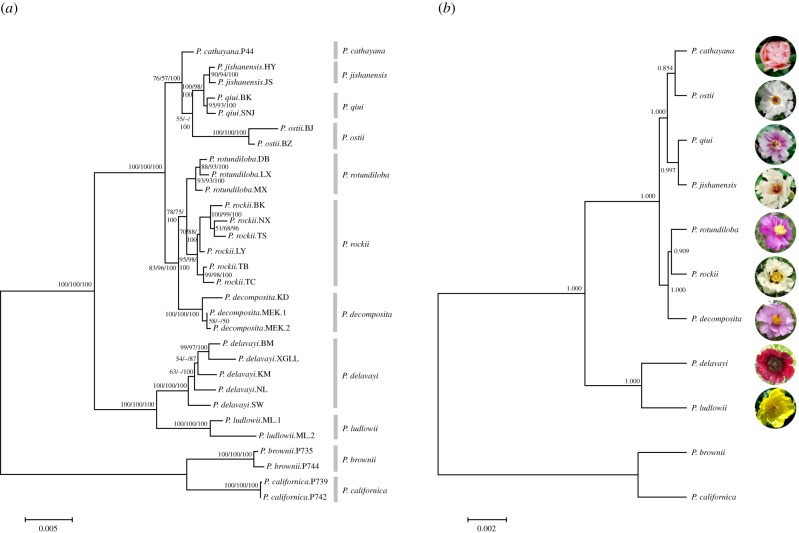
The results of phylogenetic analyses based on 25 nuclear loci from 30 individuals of nine wild tree peony species. (*a*) The ML tree inferred using PAUP from the concatenated sequences. Numbers on branches represent support values from ML/MP/BI analyses and the value below 50% is represented by a hyphen. (*b*) Species tree inferred using the *BEAST. Clade posterior probability is indicated on the branches.

When the 25 single-copy nuclear markers were analysed separately, the phylogeny of wild species was poorly resolved in the majority of the gene trees due to the lack of informative sites. We then concatenated these sequences for a combined analysis of a total of 24 521 bp sequences with 7.7% variable sites and 5.5% informative sites. The resulting phylogeny was fully resolved and each clade was well supported (figure 1*a*). All of the individuals sampled from the same species formed a monophyletic group. When rooted with *P. brownii* and *P. californica* of section *Onaepia* as the outgroups, two major branches are evident, corresponding to the two subsections of the section *Moutan*: subsect. *Delavayanae* and subsect. *Vaginatae*. In addition to the combined analyses, we inferred the species trees using *BEAST based on multispecies coalescent models (figure 1*b*). The relationship among wild species was nearly identical between the inferred species tree and the result of the combined analysis except for one difference (figure 1): *P. ostii* was sister to *P. jishanensis*/*P. qiui* on the tree resulting from the combined analysis, but was sister to *P. cathayana* in the inferred species tree. However, in either case, the sister relationship was least supported in comparison to all other clades.

A total of 14 chloroplast genes or regions that were relatively variable among the tree peony species were sampled as phylogenetic markers. A combined analysis of the 14 markers, consisting of 13 415 bp, 3.9% variable sites and 2.8% phylogenetically informative sites, resulted in a phylogeny quite different from that reconstructed based on multiple regions from the nuclear genome (electronic supplementary material, figure S1). The only relationship holds between the chloroplast and nuclear phylogenies was the monophyly of each of the two subsections. Within-species sampling failed to form monophyletic groups for four species, *P. delavayi*, *P. rotundiloba*, *P. rockii* and *P. jishanensis*. The interspecific relationships were largely incongruent with those on the nuclear tree. The two clearly separated clades within subsection *Vaginatae* in the nuclear tree, which were consistent with morphology, were intermixed in the chloroplast tree, especially with the position switched between *P. qiui*/*P. jishanensis* and *P. rotundiloba*/*P. rockii*.

**Figure 2.**
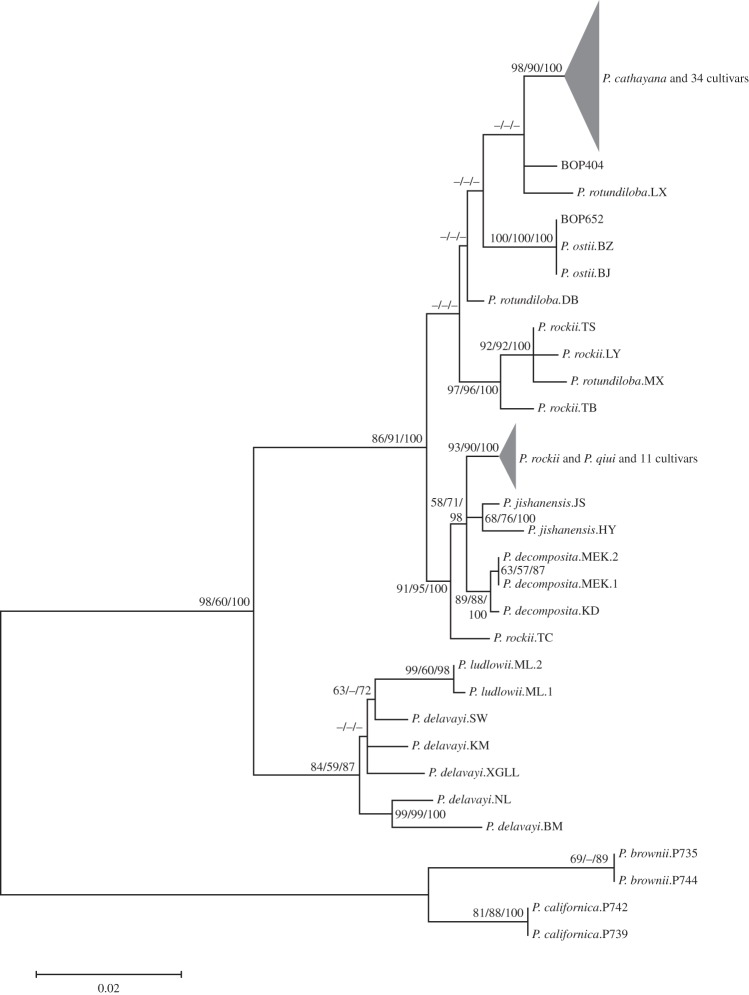
The ML tree inferred from concatenation of four chloroplast regions from wild species and traditional cultivars. Numbers on branches represent support values from ML/MP/BI analyses and the value below 50% is represented by a hyphen.

To analyse the origins of cultivated tree peonies, seven most informative nuclear regions and four most informative chloroplast regions were chosen for determining the possible parental species of the 47 sampled traditional cultivars. The combined analyses of four chloroplast markers showed that the cultivars had two major chloroplast genotypes, one shared by 34 cultivars grouped with *P. cathayana*, and the other shared by 11 cultivars grouped with *P. qiui* and certain individuals of *P. rockii* (figure 2). For the two remaining cultivars, one had the identical chloroplast sequences with *P. ostii*, and the other was related to *P. cathayana* and *P. rotundiloba*. Thus, the phylogeny suggested that *P. cathayana* served as the chloroplast donor of the majority of the cultivars and *P. qiui*/*P. rockii* was likely to be the next important chloroplast donor of the cultivated tree peonies.

**Figure 3.**
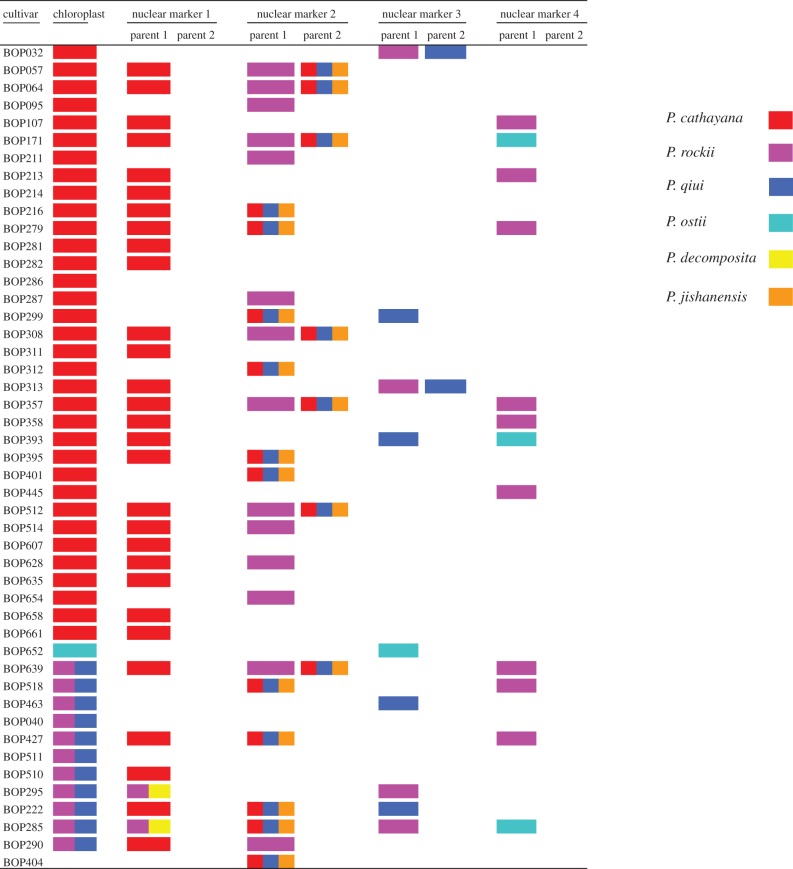
The identical sequences to wild species found in 47 traditional cultivars. For the nuclear markers, the coloured rectangles indicate that the sequence from the cultivar is 100% identical to wild species, and the multicoloured rectangles represent the situation when the sequence is identical to more than one wild species. For the chloroplast segment combined from four regions, the coloured rectangles indicate that the sequences from cultivars are nearly identical to wild species.

Of the seven nuclear makers, four were informative in terms of resolving relationships between wild species and cultivars (figure 3). For marker 1, the majority of cultivars had identical sequences to *P. cathayana*, supporting that this species served as the maternal parent for a large portion of cultivars. For the remaining three markers, however, sequences of *P. cathayana* were not dominant in the cultivars. In marker 2, sequences of *P. cathayana* were found in about half of the cultivars, but in all cases with sequences from other species. Of the species sharing sequences with cultivars in marker 2, those of *P. rockii* were found in the largest portion of the cultivars, followed by *P. cathayana*, *P. qiui* and *P. jishanensis*. For marker 3, the cultivars were found to share sequences with *P. rockii*, *P. qiui* and *P. ostii*. For marker 4, the cultivars were found to share sequences with *P. rockii* and *P. ostii*. These results suggest that the tree peony cultivars originated from hybridization between multiple wild species, including *P. cathayana*, *P. rockii*, *P. qiui*, *P. ostii* and *P. jishanensis*.

## 4. Discussion

The phylogenetic analyses of 25 nuclear markers resulted in a tree that was consistent with morphology and classification of the section *Moutan* [9,10]. Whereas the two subsections, *Delavayanae* and *Vaginatae*, are easily recognized based on morphological characters and geographical distribution, relationships and classification within the larger subsection *Vaginatae* have yet to be clarified. The nuclear gene phylogeny provided for the first time the well-resolved and highly supported phylogeny of the subsection (figure 1), which was attributed to the identification of a large number of single-copy markers with a wealth of phylogenetic information. The two major clades within the subsection are distinguished by morphological characters including the colour and height of floral discs and the number of leaflets. Within each clade, the relationships revealed here provided certain new insights. It supports the recent establishment of the species status of *P. rotundiloba*, which was once placed inside of *P. decomposita* as a susbspecies [9,10,30].

Given that the nuclear phylogeny is congruent with morphology and is very likely to represent the species tree, the chloroplast phylogeny that fails to group within-species samplings for half of the species is largely inconsistent with the species tree. This could have been a result of lineage sorting and/or natural hybridization causing chloroplast capture. Although the natural hybridization leading to the formation of allotetraploid species has been widely documented in the herbaceous section *Paeonia* of the genus [31–34], natural hybrids have not been reported in the shrubby section *Moutan*. Based on the degree of incongruence between the nuclear and chloroplast phylogenies, we speculate that introgression was not uncommon between the diploid shrubby species. Especially for the species that switch positions between the two well-supported and relatively deep-branched clades, the incongruent relationships with the nuclear gene phylogeny are more likely to have resulted from chloroplast capture. The separation of the accessions of *P. rockii* on both clades is most probably a result of natural hybridization.

To understand the origin of the cultivated tree peonies, 47 traditional cultivars were analysed with the wild species using chloroplast sequences and four most informative nuclear markers independently. The four phylogenetic markers showed different ancestral relationships of the cultivars with the wild species, demonstrating that five wild species have contributed to the origin of the cultivars most probably through hybridization. It was recorded in ancient Chinese poems that tree peonies had become very popular ornamentals more than 1000 years ago and almost every family in the ancient capital, Luoyang (of the present Henan Province), grew tree peonies in their gardens (e.g. ‘An Enumeration of Tree Peonies in Luoyang’ written by X. Ou'yang in AD 1035). It has been reported that hybridization gave rise to fertile hybrids when the wild species were brought together in household gardens [35]. Therefore, hybridization involving multiple peony species that people transplanted to their gardens could have given rise to the diverse cultivars. It was the involvement of several species with diverse morphology in the hybridization that eventually generated the amazing diversity of cultivated flowers for more than one and a half millennia.

For the majority of cultivars sampled in the study, *P. cathayana* served as the chloroplast donor or maternal parent. Several species, including *P. rockii* and *P. qiui* and *P. ostii*, were likely to be the paternal parents of these cultivars. The other group of cultivars shared the chloroplast genome of *P. qiui* together with two *P. rockii* accessions. *Paeonia cathayana* could be the primary paternal parent, while *P. ostii* seems to have also been involved in hybridization. It is possible based on the relationship of nuclear marker 2 (figure 3) that *P. jishanensis* was involved in hybridization because it shared sequences with *P. cathayana* and *P. qiui*. It is unlikely that *P. decomposita* was involved because its contribution was found only in two cultivars and the sequences were shared by *P. rockii*.

**Figure 4.**
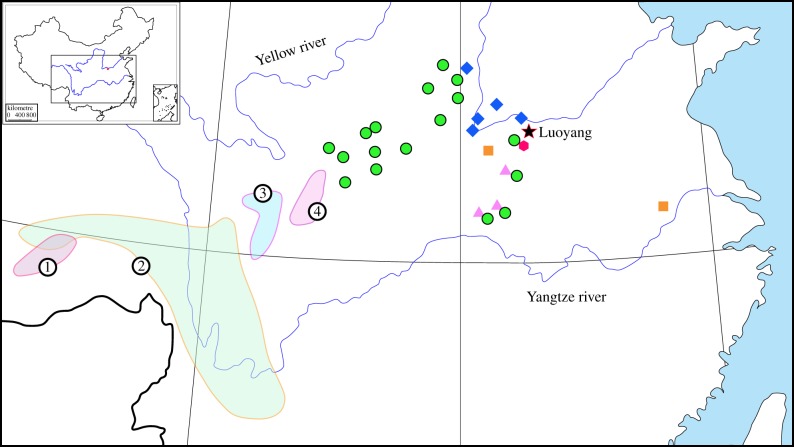
The distribution of nine wild tree peony species. (1) *Paeonia ludlowii*; (2) *P. delavayi*; (3) *P. decomposita*; (4) *P. rotundiloba*; circle, *P. rockii*; diamond, *P. jishanensis*; square, *P. ostii*; triangle, *P. qiui*; hexagon, *P. cathayana*.

The wild species involved in the origin of cultivars are all native to central China (figure 4), which was the centre of Chinese culture from the Eastern Zhou Dynasty (770 BC) to the middle Song Dynasty (AD 1126). It is intriguing to note that the majority of the wild species are rare or endangered. Most strikingly, *P. cathayana*, the maternal parent for the majority of the traditional cultivars, had only one single individual found in mountains south of Luoyang. *Paeonia ostii* also has only one individual found on a cliff in central Anhui Province. It is clear that the wild populations of the species have nearly been eradicated. *Paeonia qiui* possesses only a few small populations in western Hubei Province, while *P. jishanensis* has several relatively large populations because of its capacity of vegetative reproduction. Although *P. rockii* is still found in a relatively large area from central to western China, the populations are scattered and very small, mostly with only a few individuals.

The findings of this study have several implications. First, it is demonstrated that for the most important garden ornamental in China, its origin is a result of homoploid hybridization between five wild species with diverse flower morphology, especially in petal colours, presence or absence of purple blotches at the base of petals, and colour and height of floral discs. Second, the wild parental species of the ornamentals could have experienced drastic reduction in natural populations, and even gone endangered or extinct as a result of collection and transplanting by local people for hundreds of years. Finally, domestication of the hybrids between the wild species serves as a means of conserving at least proportions of the genomes of the endangered or extinct species. Given the fast and worldwide loss of biodiversity due to various reasons, including climate change [36], cultivars of domesticated plants and animals might be a precious gene pool maintaining genetic diversity that has already gone extinct or might be lost in the near future.

## Supplementary Material

Dataset S1

## Supplementary Material

Figure S1

## Supplementary Material

Table S1

## Data accessibility

DNA sequences generated for this study have been deposited in the GenBank database under accession numbers KM092534–KM093728 and KJ945637–KJ946200.
