# Supplementary material for: Multiple species of wild tree peonies gave rise to the ‘king of flowers’, Paeonia suffruticosa Andrews
Source: Proc Biol Sci. 2014 Dec 22;281(1797):20141687. doi: 10.1098/rspb.2014.1687 (PMC4240985; doi:10.1098/rspb.2014.1687)
Supplement: Figure S1 [file rspb20141687supp2.pdf]

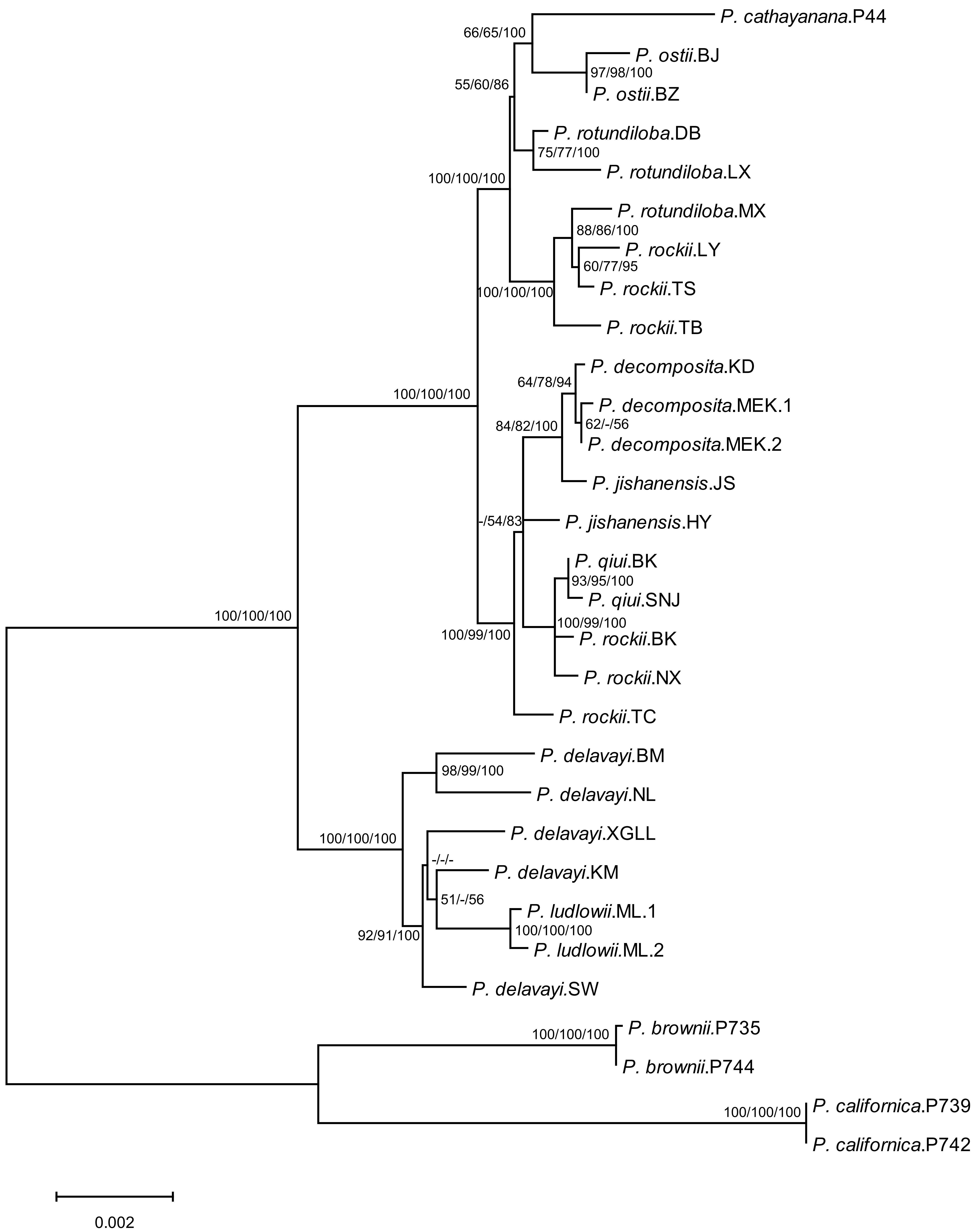

Figure S1. The maximum likelihood tree inferred from concatenation of 14 chloroplast regions from wild species. Numbers on braches represent support values from ML/MP/BI analyses and the value below 50% is represented by hyphen.
