# Supplementary material for: Multiple species of wild tree peonies gave rise to the ‘king of flowers’, Paeonia suffruticosa Andrews
Source: Proc Biol Sci. 2014 Dec 22;281(1797):20141687. doi: 10.1098/rspb.2014.1687 (PMC4240985; doi:10.1098/rspb.2014.1687)
Supplement: Table S1 [file rspb20141687supp3.doc]

**Table S1**. Materials sampled in this study, including nine wild species of sect. *Moutan*, two species of sect. *Onaepia* which used as outgroups, and 47 cultivars.

| Category | Taxon | Author of the scientific name of a taxon / Cultivar name |  | Country | Province | City or county | Symbol in figures |
| --- | --- | --- | --- | --- | --- | --- | --- |
| Ingroup | *Paeonia cathayana* | D. Y. Hong & K. Y. Pan |  | China | Henan | Songxian County | *P.cathayana*.P44 |
| Ingroup | *Paeonia decomposita* | Hand.-Mazz. |  | China | Sichuan | Kangding City | *P.decomposita.*KD |
| Ingroup | *Paeonia decomposita* | Hand.-Mazz. |  | China | Sichuan | Markam Town | *P.decomposita.*MEK.1 |
| Ingroup | *Paeonia decomposita* | Hand.-Mazz. |  | China | Sichuan | Songgan Town | *P.decomposita.*MEK.2 |
| Ingroup | *Paeonia delavayi* | Franch. |  | China | Tibet | Bomi County | *P.delavayi*.BM |
| Ingroup | *Paeonia delavayi* | Franch. |  | China | Yunnan | Ninglang County | *P.delavayi.*NL |
| Ingroup | *Paeonia delavayi* | Franch. |  | China | Yunnan | Shangri-La | *P.delavayi.*XGLL |
| Ingroup | *Paeonia delavayi* | Franch. |  | China | Sichuan | Muli County | *P.delavayi.*SW |
| Ingroup | *Paeonia delavayi* | Franch. |  | China | Yunnan | Kunming City | *P.delavayi.*KM |
| Ingroup | *Paeonia jishanensis* | T. Hong & W. Z. Zhao |  | China | Shaanxi | Huaying County | *P.jishanensis.*HY |
| Ingroup | *Paeonia jishanensis* | T. Hong & W. Z. Zhao |  | China | Shanxi | Jishan County | *P.jishanensis.*JS |
| Ingroup | *Paeonia ludlowii* | (Stern & G. Taylor) D. Y. Hong |  | China | Tibet | Mailin County | *P.ludlowii*.ML.1 |
| Ingroup | *Paeonia ludlowii* | (Stern & G. Taylor) D. Y. Hong |  | China | Tibet | Mailin County | *P.ludlowii.*ML.2 |
| Ingroup | *Paeonia ostii* | T. Hong & J. X. Zhang |  | China | Beijing | Beijing | *P.ostii.*BJ |
| Ingroup | *Paeonia ostii* | T. Hong & J. X. Zhang |  | China | Anhui | Bozhou City | *P.ostii*.BZ |
| Ingroup | *Paeonia qiui* | Y. L. Pei & D. Y. Hong |  | China | Hubei | Baokang County | *P.qiui*.BK |
| Ingroup | *Paeonia qiui* | Y. L. Pei & D. Y. Hong |  | China | Hubei | Shennongjia | *P.qiui.*SNJ |
| Ingroup | *Paeonia rockii* | (S. G. Haw & Lauener) T. Hong & J. J. Li ex D. Y. Hong |  | China | Hubei | Baokang County | *P.rockii.*BK |
| Ingroup | *Paeonia rockii* | (S. G. Haw & Lauener) T. Hong & J. J. Li ex D. Y. Hong |  | China | Shaanxi | Lueyang County | *P.rockii.*LY |
| Ingroup | *Paeonia rockii* | (S. G. Haw & Lauener) T. Hong & J. J. Li ex D. Y. Hong |  | China | Henan | Neixiang County | *P.rockii.*NX |
| Ingroup | *Paeonia rockii* | (S. G. Haw & Lauener) T. Hong & J. J. Li ex D. Y. Hong |  | China | Shaanxi | Tongchuan County | *P.rockii.*TC |
| Ingroup | *Paeonia rockii* | (S. G. Haw & Lauener) T. Hong & J. J. Li ex D. Y. Hong |  | China | Shaanxi | Taibai County | *P.rockii.*TB |
| Ingroup | *Paeonia rockii* | (S. G. Haw & Lauener) T. Hong & J. J. Li ex D. Y. Hong |  | China | Gansu | Tianshui City | *P.rockii.*TS |
| Ingroup | *Paeonia roundiloba* | (D. Y. Hong) D. Y. Hong |  | China | Gansu | Diebu County | *P.roundiloba.*DB |
| Ingroup | *Paeonia roundiloba* | (D. Y. Hong) D. Y. Hong |  | China | Sichuan | Lixian County | *P.roundiloba.*LX |
| Ingroup | *Paeonia roundiloba* | (D. Y. Hong) D. Y. Hong |  | China | Sichuan | Maoxian County | *P.roundiloba.*MX |
| Outgroup | *Paeonia brownii* | Douglas ex Hook. |  | USA | Oregon | Wallowa County | *P.brownii.*P735 |
| Outgroup | *Paeonia brownii* | Douglas ex Hook. |  | USA | Oregon | Union County | *P.brownii.*P744 |
| Outgroup | *Paeonia californica* | Nutt. ex Torr. & A. Gray |  | USA | California | San Bernardino | *P.califonica.*P739 |
| Outgroup | *Paeonia californica* | Nutt. ex Torr. & A. Gray |  | USA | California | Los Angeles County | *P.califonica.*P742 |
| Cultivar | *Paeonia suffruticosa* | ‘Ba Bao Xiang’ |  | China | Henan | Luoyang | BOP286 |
| Cultivar | *Paeonia suffruticosa* | ‘Bai Yu’ |  | China | Henan | Luoyang | BOP107 |
| Cultivar | *Paeonia suffruticosa* | ‘Bang Ning Zi’ |  | China | Shandong | Hezhe | BOP658 |
| Cultivar | *Paeonia suffruticosa* | ‘Chun Hong Zheng Yan’ |  | China | Shandong | Hezhe | BOP518 |
| Cultivar | *Paeonia suffruticosa* | ‘Da Hong Jian Rong’ |  | China | Shandong | Hezhe | BOP661 |
| Cultivar | *Paeonia suffruticosa* | ‘Di Yi Jiao’ |  | China | Shandong | Hezhe | BOP514 |
| Cultivar | *Paeonia suffruticosa* | ‘Dou Lü’ |  | China | Henan | Luoyang | BOP393 |
| Cultivar | *Paeonia suffruticosa* | ‘Er Qiao’ |  | China | Henan | Luoyang | BOP295 |
| Cultivar | *Paeonia suffruticosa* | ‘Feng Dan Bai’ |  | China | Henan | Luoyang | BOP652 |
| Cultivar | *Paeonia suffruticosa* | ‘Ge Jin Zi’ |  | China | Henan | Luoyang | BOP395 |
| Cultivar | *Paeonia suffruticosa* | ‘Hai Tan Zheng Run’ |  | China | Henan | Luoyang | BOP463 |
| Cultivar | *Paeonia suffruticosa* | ‘Hei Hua Kui’ |  | China | Henan | Luoyang | BOP282 |
| Cultivar | *Paeonia suffruticosa* | ‘Hu Hong’ |  | China | Henan | Luoyang | BOP311 |
| Cultivar | *Paeonia suffruticosa* | ‘Huang Hua Kui’ |  | China | Henan | Luoyang | BOP095 |
| Cultivar | *Paeonia suffruticosa* | ‘Jiao Rong San Bian’ |  | China | Henan | Luoyang | BOP287 |
| Cultivar | *Paeonia suffruticosa* | ‘Jin Lun Huang’ |  | China | Henan | Luoyang | BOP628 |
| Cultivar | *Paeonia suffruticosa* | ‘Jin Yu Jiao Zhang’ |  | China | Henan | Luoyang | BOP213 |
| Cultivar | *Paeonia suffruticosa* | ‘Jin Zhang Fu Rong’ |  | China | Henan | Luoyang | BOP312 |
| Cultivar | *Paeonia suffruticosa* | ‘Jiu Zui Yang Fei’ |  | China | Henan | Luoyang | BOP171 |
| Cultivar | *Paeonia suffruticosa* | ‘Lan Tian Yu’ |  | China | Henan | Luoyang | BOP358 |
| Cultivar | *Paeonia suffruticosa* | ‘Luo Yang Hong’ |  | China | Henan | Luoyang | BOP285 |
| Cultivar | *Paeonia suffruticosa* | ‘Mo Kui’ |  | China | Henan | Luoyang | BOP222 |
| Cultivar | *Paeonia suffruticosa* | ‘Mo Sha Jin’ |  | China | Henan | Luoyang | BOP214 |
| Cultivar | *Paeonia suffruticosa* | ‘Pan Zhong Qu Guo’ |  | China | Henan | Luoyang | BOP032 |
| Cultivar | *Paeonia suffruticosa* | ‘Po Mo Zi’ |  | China | Henan | Luoyang | BOP057 |
| Cultivar | *Paeonia suffruticosa* | ‘Qie Lan Dan Sha’ |  | China | Henan | Luoyang | BOP308 |
| Cultivar | *Paeonia suffruticosa* | ‘Qin Hong’ |  | China | Henan | Luoyang | BOP639 |
| Cultivar | *Paeonia suffruticosa* | ‘Qing Long Wo Mo Chi’ |  | China | Henan | Luoyang | BOP607 |
| Cultivar | *Paeonia suffruticosa* | ‘Qing Shan Guan Xue’ |  | China | Henan | Luoyang | BOP445 |
| Cultivar | *Paeonia suffruticosa* | ‘San Qi Ji Sheng’ |  | China | Henan | Luoyang | BOP654 |
| Cultivar | *Paeonia suffruticosa* | ‘Shi Ba Hao’ |  | China | Henan | Luoyang | BOP279 |
| Cultivar | *Paeonia suffruticosa* | ‘Shou An Hong’ |  | China | Henan | Luoyang | BOP313 |
| Cultivar | *Paeonia suffruticosa* | ‘Song Bai’ |  | China | Shandong | Hezhe | BOP357 |
| Cultivar | *Paeonia suffruticosa* | ‘Tao Hong Xian Mei’ |  | China | Shandong | Hezhe | BOP511 |
| Cultivar | *Paeonia suffruticosa* | ‘Wei Zi’ |  | China | Henan | Luoyang | BOP299 |
| Cultivar | *Paeonia suffruticosa* | ‘Wen Gong Hong’ |  | China | Shandong | Hezhe | BOP512 |
| Cultivar | *Paeonia suffruticosa* | ‘Wu Long Peng Sheng’ |  | China | Henan | Luoyang | BOP281 |
| Cultivar | *Paeonia suffruticosa* | ‘Xiang Yang Da Hong’ |  | China | Shandong | Hezhe | BOP510 |
| Cultivar | *Paeonia suffruticosa* | ‘Yan Long Zi Zhu Pan’ |  | China | Shandong | Hezhe | BOP401 |
| Cultivar | *Paeonia suffruticosa* | ‘Yao Huang’ |  | China | Henan | Luoyang | BOP635 |
| Cultivar | *Paeonia suffruticosa* | ‘Yi Pin Zhu Yi’ |  | China | Henan | Luoyang | BOP290 |
| Cultivar | *Paeonia suffruticosa* | ‘Yin Luo Bao Zhu’ |  | China | Henan | Luoyang | BOP040 |
| Cultivar | *Paeonia suffruticosa* | ‘Zhang Hua An’ |  | China | Shandong | Hezhe | BOP404 |
| Cultivar | *Paeonia suffruticosa* | ‘Zhao Fen’ |  | China | Henan | Luoyang | BOP427 |
| Cultivar | *Paeonia suffruticosa* | ‘Zhi Hong’ |  | China | Henan | Luoyang | BOP211 |
| Cultivar | *Paeonia suffruticosa* | ‘Zhong Sheng Hong’ |  | China | Henan | Luoyang | BOP216 |
| Cultivar | *Paeonia suffruticosa* | ‘Zhuang Yuan Hong’ |  | China | Henan | Luoyang | BOP064 |
